# Changes in resting‐state functional connectivity in nonacute sciatica with acupuncture modulation: A preliminary study

**DOI:** 10.1002/brb3.1494

**Published:** 2020-01-10

**Authors:** Ching‐Hsiung Liu, Tzu‐Chen Yeh, Yen‐Ying Kung, Hung‐Pin Tseng, Ching‐Ju Yang, Tzu‐Yi Hong, Chou‐Ming Cheng, Jen‐Lin Yang, Ta‐Peng Wu, Jen‐Chuen Hsieh, Fang‐Pey Chen

**Affiliations:** ^1^ Department of Neurology Lotung Poh‐Ai Hospital Ilan Taiwan; ^2^ Institute of Traditional Medicine School of Medicine National Yang‐Ming University Taipei Taiwan; ^3^ Integrated Brain Research Unit Department of Medical Research and Education Taipei Veterans General Hospital Taipei Taiwan; ^4^ Institute of Brain Science School of Medicine National Yang‐Ming University Taipei Taiwan; ^5^ Department of Radiology Taipei Veterans General Hospital Taipei Taiwan; ^6^ Center for Traditional Medicine Taipei Veterans General Hospital Taipei Taiwan; ^7^ Faculty of Medicine School of Medicine Yang‐Ming University Taipei Taiwan; ^8^ Brain Research Center National Yang‐Ming University Taipei Taiwan

**Keywords:** acupuncture, default mode network, functional connectivity, magnetic resonance imaging, sciatica

## Abstract

**Aims:**

To investigate the functional connectivity (FC) in nonacute sciatica and the neuronal correlation of acupuncture analgesia.

**Methods:**

A prospective study employing resting‐state functional magnetic resonance imaging was conducted. Twelve sciatica patients were enrolled to receive six or 18 acupoints of acupuncture treatment twice a week for 4 weeks. Regional homogeneity (ReHo) and seed‐based FC were performed.

**Results:**

Regional homogeneity analysis demonstrated a greater alteration in the right posterior cingulate cortex (PCC) during the pre‐acupuncture phase than during the postacupuncture phase. Compared to that of healthy controls, the PCC‐seeded FC (default mode network, DMN) of sciatica patients exhibited hyperconnectivity of PCC‐FC with the PCC‐bilateral insula, cerebellum, inferior parietal lobule, right medial prefrontal cortex, and dorsal anterior cingulate cortex during the pre‐acupuncture phase as well as hypoconnectivity of PCC‐FC with the right cerebellum, left precuneus, and left dorsal medial prefrontal cortex during the postacupuncture phase. Correlation analysis between PCC‐seeded FC and behavior measurements revealed a positive association with the duration of sciatica in the right inferior parietal lobule prior to acupuncture treatment.

**Conclusions:**

Acupuncture in chronic sciatica patients is associated with normalized DMN activity and modulation of descending pain processing. The changes in the subclinical endophenotype of brain FC after acupuncture treatment may provide clues for understanding the mechanism of acupuncture‐mediated analgesia in chronic pain.

## Graphical abstract

Acupuncture modulation elicited a bidirectionally regulated effect on default mode network (DMN) in chronic sciatica patients. The changes in subclinical endophenotype of brain FC after acupuncture treatment may provide clues for understanding the mechanism of acupuncture‐mediated analgesia in chronic pain.

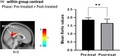

## 1 INTRODUCTION

Sciatica is a common chronic pain disorder, typically presenting as low back or gluteal pain that may radiate to one leg, with motor or sensory complaints (Peul et al., 2007; Valat, Genevay, Genevay, Marty, Rozenberg, & Koes, 2010). A measurement of the global burden of disease revealed that low back pain is the leading cause of daily disability, and sciatica is one of the most common variations of low back pain (Andersson, Pope, Frymoyer, & Snook, 1991; Murray & Lopez, 2013). The prevalence of sciatica varies from 1.6% up to 43% in the population aged 20 years and older, highlighting its ununified diagnostic criteria and complex manifestations (Deyo & Tsui‐Wu, 1987; Videman et al., 1984).

A systemic review and meta‐analysis of drugs for pain relief of sciatica reported that management of sciatica in primary care is still unclear (Pinto et al., 2012), and the effectiveness of physical therapy, bed rest, manipulation, or medication could not be concluded from previous literature (Luijsterburg et al., 2007). Network meta‐analyses comparing the clinical effectiveness of management strategies for sciatica demonstrate that for overall outcomes, there is a statistically significant improvement following disk surgery, epidural injections, nonopioid analgesia, manipulation, and acupuncture (Lewis et al., 2015).

Chronic pain is a complex negative experience that engages multiple regions of the brain (Apkarian, Baliki, Baliki, & Geha, 2009). Chronic sciatica is classified as either persistent or multiple acute recurrences (Atlas & Nardin, 2003). Therefore, pain‐induced disability may affect sciatica patients. The key pathway of pain indicates the transmission of nociception to our brain. Many functional magnetic resonance imaging (fMRI) studies have identified that chronic pain may cause changes in the brain (May, 2008).

A recent study indicated that the interaction between large‐scale brain networks could be dynamically changed in patients with chronic pain (Kucyi & Davis, 2015). The default mode network (DMN) is a crucial brain network composed of regions including the posterior cingulate cortex/precuneus (PCC/PCu), medial prefrontal cortex (mPFC), inferior parietal cortex, inferior temporal cortex, and hippocampal cortex (Buckner, Andrews‐Hanna, Andrews‐Hanna, & Schacter, 2008). The DMN typically exhibits more activity during rest than during task engagement (Raichle, 2015). DMN alterations have been reported in numerous neuropsychiatric diseases and chronic pain disorder (Baliki, Mansour, Mansour, Baria, & Apkarian, 2014; Farmer et al., 2011). Aberrant functional connectivity (FC) of the DMN may also contribute to spontaneous neuropathic pain (Cauda et al., 2009).

Resting‐state fMRI (rsfMRI) findings showed that acupuncture may modulate the FC of the brain (Chen et al., 2015; Jia et al., 2015; Shi et al., 2015). Acupuncture may achieve its therapeutic effect on knee osteoarthritis pain by modulating FC between the right frontoparietal network, executive control network (ECN), and descending pain modulatory pathway (Chen et al., 2015). In an acute experimental low back pain study, compared with the baseline, the pain state had higher regional homogeneity (ReHo) values in the pain matrix, limbic system, and DMN; acupuncture produced broad deactivation in the brain, including the limbic system, pain status, and DMN (Shi et al., 2015). The central pain relief mechanism of acupuncture is being increasingly elucidated in recent years. A meta‐analysis of 149 studies revealed greater deactivation in the subgenual anterior cingulate, subgenual cortex, amygdala/hippocampal formation, ventromedial PFC, nucleus accumbens, and PCC during acupuncture (Huang et al., 2012). A review article on brain FC network studies proposed that acupuncture effects may increase FC of DMN and sensorimotor network with pain, as well as affective and memory‐related brain regions (Cai, Shen, Shen, Wang, & Guan, 2018).

We hypothesized that if acupuncture therapy is effective for pain relief in nonacute/chronic sciatica, changes in the activity of a key network related to acupuncture modulation, such as the DMN, may be observed (Dhond, Yeh, Yeh, Park, Kettner, & Napadow, 2008). ReHo analysis is used to estimate the local coherence of spontaneous brain activity (Zang, Jiang, Jiang, Lu, He, & Tian, 2004). ReHo seed‐based FC analysis may bypass seed‐selection ambiguity and improve the sensitivity in detecting FC of the DMN or task‐positive network (Yan et al., 2013). Further, changes in ReHo can measure the local FC, which can be used to detect brain regions with abnormal local FC caused by lesions (Jiang & Zuo, 2016). In the present study, we performed seed‐based FC analysis based on the ReHo method to explore the activity patterns of the DMN in sciatica patients compared with healthy controls, and the central analgesic effect of acupuncture in treating chronic sciatica.

## 2 METHODS

### 2.1 Participants

Subjects who met the inclusive and diagnostic criteria were recruited for this study via the outpatient department. Twelve patients and 15 healthy people were recruited. Informed consent was obtained from all individual participants included in the study, and the study was approved by the Institutional Review Board of Taipei Veterans General Hospital.

All patients who met the following inclusion criteria were screened: (a) 35–85 years old, (b) low back or gluteal pain radiating into one leg, and (c) pain duration of at least 2 weeks. Exclusion criteria were (a) known or suspected serious spinal pathology (e.g., cauda equina syndrome or spinal fracture), (b) pregnant or breast‐feeding women, (c) scheduled or being considered for spinal surgery or interventional procedures for sciatica during the 8‐week treatment period, (d) administration of sedative or analgesic drugs within 24 hr before the fMRI scan, (e) comorbidities of systemic malignancy, bleeding tendency, rheumatic arthritis, and other known autoimmune diseases, (f) focal neurological deficits with progressive or disabling symptoms, (g) history of receiving acupuncture treatment in the past 1 month, (h) contraindications for acupuncture or MRI, (i) visual analog scale <3, and (j) low back pain without sciatica.

In addition to inclusion and exclusion criteria, the patient's diagnosed sciatica met at least two of the following items: (a) radicular pain in the L4, L5, or S1 dermatome; (b) physical examination consistent with radicular pain, motor or sensory neurological findings, or decreased reflex in the L4, L5, or S1 nerve root distribution; (c) positive straight‐leg‐raising test; (d) increased leg pain on coughing, sneezing, or straining; and (e) MRI demonstrating a unilateral disk herniation impinging on the L4, L5, or S1 nerve root (Table 1).

**Table 1:** Demographic data at baseline and after acupuncture modulation

Characteristics	Before acupuncture (*n* = 12)	After acupuncture (*n* = 12)	*p*‐Value
Gender (M/F)	6/6	6/6	—
Age (years)	61.42 ± 14.84	—	—
Duration (months)	20.75 ± 16.44	—	—
Side (right/left)	6/6	—	
VAS	5.42 ± 1.83	3.92 ± 1.83	.032*
SBI	11.83 ± 5.37	9.42 ± 4.10	.287
RDQS	10.75 ± 4.85	7.17 ± 5.04	.036*
WHOQOL‐BREF, total	53.19 ± 9.48	52.68 ± 8.28	.827
Physical	12.68 ± 2.54	13.34 ± 1.87	.388
Psychological	13.17 ± 2.55	12.91 ± 2.07	.651
Social	13.58 ± 3.09	12.83 ± 3.46	.298
Environmental	13.67 ± 2.61	13.41 ± 1.92	.660

Note

Data are presented as mean ± *SD* and were compared using independent *t* test (continuous variables).

Abbreviations: RDQS, Roland Disability Questionnaire for Sciatica; SBI, Sciatica Bothersomeness Index; VAS, visual analog scale; WHOQOL‐BREF, the World Health Organization Quality of Life in the Brief Edition.

* *p* < .05; **p < .01.

### 2.2 Study design

A prospective observational study was performed. The study comprised two arms: an acupuncture group and a healthy control group. Prior to acupuncture treatment (week 0, baseline) and after 4 weeks of treatment (eight sessions), rsfMRI scans were obtained. The total observation period in this study was 4 weeks for each patient. All patients received brain fMRI and behavioral scales twice (at baseline and 4 weeks after acupuncture treatment). The healthy individuals received one fMRI and behavior scale only, without any intervention.

We reviewed previous acupuncture treatment for sciatica in MEDLINE (PubMed) since 1970 and selected acupoints that were effective for treatment (Liu & Chen, 2017). We chose to use a combined acupoint treatment strategy, considering that treatment using acupoint combinations may achieve a synergistic effect in the brain (Zhang et al., 2019, 2016). All patients with sciatica received either six or 18 acupoints of manual acupuncture. The six acupoints were Shenshu (BL 23), Huantiao (GB 30), Weizhong (BL 40), Yanglingquan (GB 34), Kunlun (BL 60), and Xuanzhong (GB 39). The eighteen acupoints were Shenshu (BL 23), Dachangshu (BL 25), Xiaochangshu (BL 27), Huantiao (GB 30), Yinmen (BL 37), Zhibian (BL 54), Chengfu (BL 36), Fengshi (GB 31), Weizhong (BL 40), Zusanli (ST 36), Yanglingquan (GB 34), Yinlingquan (SP 9), Feiyang (BL 58), Sanyinjiao (SP 6), Xuanzhong (GB 39), Kunlun (BL 60), Taixi (KI 3), and Shenmai (BL 62). The acupoints selected were primarily in the BL and GB meridians, plus the governing vessels and KI meridians, which may nourish the patient's *qi*, based on the theory of Traditional Chinese Medicine (Qin, Liu, Liu, Wu, Zhai, & Liu, 2015).

### 2.3 Intervention

The assessment of sciatica was conducted by a neurologist with 20 years of experience in neurology. Acupuncture treatment was administered by a qualified acupuncturist who has been practicing in the Department of Traditional Chinese Medicine for over 15 years.

Sterile, disposable, 0.3 × 40 mm or 0.35 × 50 mm (diameter × length) stainless steel needles (Ching Ming Medical Device Co) were used. Needles were only used once and were generally inserted to a depth of 5–30 mm, depending on the acupuncture point. Insertion was followed by manual stimulation to elicit the “de qi” sensation. Needles were retained in position for 20 min of acupuncture treatment. The acupuncture treatment was provided twice weekly for 4 weeks in both groups (i.e., eight treatments in total).

### 2.4 Clinical outcome measures

The primary outcome was the 100‐mm visual analog scale (VAS) for sciatica, measured at baseline and week 4. The pain scores ranged from 0 to 10; higher values represented worse outcomes. The patients were asked to rate their average leg pain over the last 24 hr with 0 representing “no leg pain” and 10 representing the “worst pain imaginable” (Collins, Moore, Moore, & McQuay, 1997; Huskisson, 1974).

The major secondary outcome was the Roland Disability Questionnaire for Sciatica (RDQS; Kim, Guilfoyle, Seeley, & Laing, 2010; Roland & Morris, 1983) measured at baseline and week 4 to assess functional disability (score 0: no disability; score 24: most disability); higher values represent worse outcomes. Sciatica Bothersomeness Index (SBI) was introduced to rate leg pain, as well as other bothersome symptoms such as paresthesia and weakness (score 0: not bothersome; score 24: extremely bothersome). Higher symptom scores were associated with higher emotional distress and lower physical function (Grovle et al., 2010; Grovle, Haugen, Haugen, Natvig, Brox, & Grotle, 2013). Further, the World Health Organization Quality of Life in the Brief Edition (WHOQOL‐BREF) was scored at baseline and week 4 for evaluating medical outcomes and quality of life; higher scores indicate better quality of life. There were four subscales: (a) physical health, score 4–20; (b) psychological, score 4–20; (c) social relationships, score 4–20; and (d) environment, score 4–20. The total score had a minimum of 8 and maximum of 80; higher values represent better outcomes (Yao & Wu, 2005).

### 2.5 Image acquisition

Resting‐state fMRI scans were obtained from each group at baseline and after 4 weeks of treatment to detect the regional features of spontaneous brain activity. Resting‐state fMRI was performed with a 3.0‐Tesla scanner (Discovery MR750; GE Healthcare) and 12‐channel head coil. Echo‐planar imaging was used for the rsfMRI scans (repetition number = 200 for conventional echo‐planar imaging; dummy scan = 5; repetition time/echo time = 2,000/30 ms; flip angle = 90°; matrix size = 64 × 64 × 40; and slice number = 40, slice thickness 3 mm, and field of view = 230 × 230 mm^2^). With eye fixation after correction of visual acuity, the baseline study of resting brain was obtained with (a) 5‐min sensory deprivation after explaining the resting condition to subjects and (b) subject's response at the end of the scan, verifying their state of consciousness. An online real‐time analysis of head motion, using the methods modified from Analysis of Functional Neuroimaging (NIMH), ensured the quality of fMRI images with head translation <1 mm and head rotation <0.5° within each session. Studies of head motion exceeding the motion criteria were repeated or rejected from data analysis. Structural MRI studies included (1) 3D magnetization‐preparation T1‐weighted structural image (repetition time/echo time/inversion time = 8.2/3.2/450 ms, flip angle = 12°, matrix size = 256 × 256 × 176, field of view = 230 × 230 mm^2^, voxel size = 0.9 × 0.9 × 0.9 mm^3^). The total scan time of the rsfMRI was approximately 13 min. Head cushions and earplugs were provided to reduce head motion and noise. During the fMRI examination, the patient's vital signs such as heart rate, O_2_ saturation, and respiratory rate were monitored and recorded.

### 2.6 Analysis of resting‐state functional MRI data

#### 2.6.1 Image preprocessing

Preprocessing was performed using the Data Processing Assistant for Resting‐State fMRI V2.3 basic edition (State Key Laboratory of Cognitive Neuroscience and Learning, Beijing Normal University, China; Chao‐Gan & Yu‐Feng, 2010) with Statistical Parametrical Mapping 8 (SPM8; Wellcome Trust Center for Neuroimaging, University College London, London, UK) in MATLAB 2014a (The MathWorks, Inc.). Functional image data were corrected for any head movements using SPM‐realign, a linear transformation procedure. We coregistered structural data with SPM coregister and normalized it to the Montreal Neurological Institute (MNI) space using a standard MNI template (SPM‐normalize) and then smoothed it with SPM‐smooth methods.

#### 2.6.2 Imaging postprocessing: ReHo analysis

ReHo was used for data‐driven and regions of interest as seed‐base analysis. ReHo maps were created by the following analysis (Zang et al., 2004): The resulting time series in each voxel was then linearly detrended and band‐pass‐filtered (0.01–0.08 Hz) to extract the low‐frequency oscillations. The following nuisance variables were regressed out: (a) the six head movement parameters computed based on rigid body translation and rotation during the realignment in SPM8; (b) the mean signal within the lateral ventricles of cerebrospinal fluid; and (c) the mean signal within a deep white matter region (centrum ovale). Brain activity was activated in clusters or contiguous voxels. ReHo maps were generated by calculating Kendall's coefficient of concordance of the time series between a given voxel with its nearest neighbors (26 voxels) in a voxel‐wise manner. The ReHo maps were spatially smoothed using a 3D Gaussian kernel of 8 mm full‐width at half‐maximum. For standardization purposes, each individual subject's ReHo map was divided by its own global mean. The peaks of significant clusters were then selected as the ReHo‐based seeds.

#### 2.6.3 Functional connectivity analysis of ReHo‐based seeds

The preprocessing of the FC analysis protocol was the same as that of the ReHo analysis, except that the images were (a) coregistered to individuals' anatomical image and then normalized to the standard T1 MNI template; (b) spatially smoothed using a 3D Gaussian kernel of 8 mm full‐width at half‐maximum; and (c) treated with the global mean signal for additional nuisance variables. Because the global signal regression may cause a negative shift in the distribution of correlations (Fox, Zhang, Zhang, Snyder, & Raichle, 2009; Murphy, Birn, Birn, Handwerker, Jones, & Bandettini, 2009), a positive mask was implemented to address positive connectivity only. The seeded regions that were selected for FC analysis were those wherein the ReHo significantly differed in the between‐group (compared with controls) and within‐group (before and after treatment) comparisons. The mean time‐series activity was extracted within the 3‐mm‐radius spherical ReHo‐seeded regions. The individual FC maps were computed by Pearson's correlation coefficient (*r*) between the seeds and the related brain regions. After calculating the correlation between the reference time course and the time course of each voxel in the brain, *r*‐values were converted into *z*‐values using Fisher's *r*‐to‐*z* transformation to normalize the distribution. The FC was considered significant if the family‐wise error level was *p* < .05 at the voxel level.

### 2.7 Statistical analysis

#### 2.7.1 Demographic and behavior data

SPSS statistical package program (version 19.0; SPSS Inc.) was used. The significance level used for the statistical analysis with two‐tailed testing was 5%. Continuous variables were presented as means ± standard deviation with 95% confidence intervals (CI). Categorical variables were described as *n* (%). Treatment effects such as 100 mm VAS, SBI, RDQS, and WHOQOL‐BREF, measured at baseline and week 4, were evaluated using a paired *t* test for within‐group analysis.

#### 2.7.2 Image data: ReHo and ReHo‐seeded FC

One‐sample *t* tests were performed to derive group maps of FC (ReHo‐seeded FC), paired *t* tests were performed to compare the changes in FC before and after acupuncture in patients with sciatica, and independent‐sample *t* tests were performed to compare changes in FC in the patients with sciatica and healthy controls. A cluster‐corrected *p* < .05 (family‐wise error correction) level was used as the threshold for statistical significance.

#### 2.7.3 Correlation analyses

Between ReHo‐seeded FC and behavior, data were analyzed using a one‐sample *t* test to examine the correlation between behavior inventories (VAS, SBI, and RDQS) and baseline in patients with sciatica. Two‐sample *t* tests were conducted to determine the correlation between behavior inventories and ReHo‐seeded FC in the acupuncture group for the condition of before and after treatment, respectively. Significance was set at the uncorrected voxel level *p* < .005, followed by the family‐wise error‐corrected cluster level *p* < .05.

## 3 RESULTS

### 3.1 Baseline information and demographic data

Twelve patients (six male) completed the study. Each patient received two fMRI scans (one at baseline and one after eight sessions of acupuncture at 4 weeks). The average ages of patients were 61.42 ± 14.84 years. The mean disease duration was 20.75 ± 16.44 months. Six patients had right sciatica, and six patients had left sciatica. The mean VAS, SBI, RDQS, and total WHOQOL‐BREF at baseline were 5.42 ± 1.83, 11.83 ± 5.37, 10.75 ± 4.85, and 53.19 ± 9.48, respectively (Table 1).

### 3.2 Measurements of pain and quality of life

**Figure 1:**
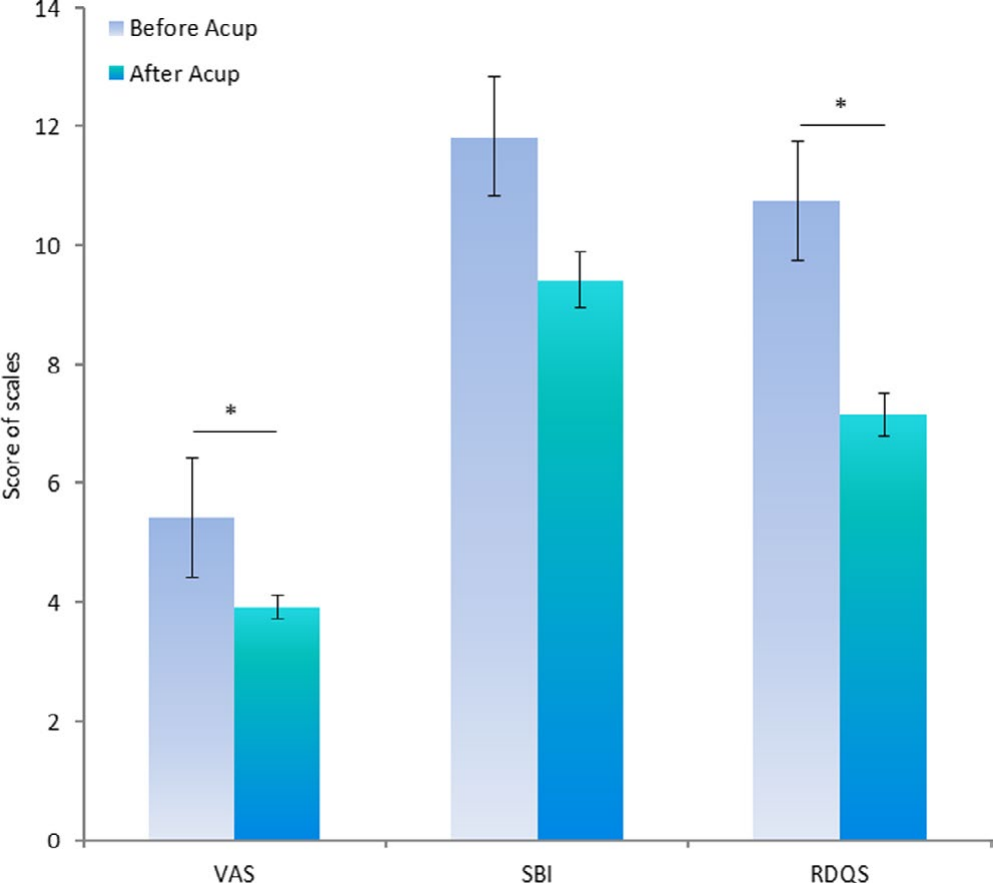
Behavior scales before and after acupuncture treatment. Acup, acupuncture; RDQS, Roland Disability Questionnaire for Sciatica; SBI, Sciatica Bothersomeness Index; VAS, visual analog scale. *p < .05

There were significant differences between before and after acupuncture treatment with regard to VAS and RDQS (*p* = .032 and .036, respectively). However, there were nonsignificant changes observed in SBI and WHOQOL‐BREF after acupuncture treatment (Table 1 and Figure 1).

### 3.3 Intra‐ and interregional connectivity of ReHo

**Figure 2:**
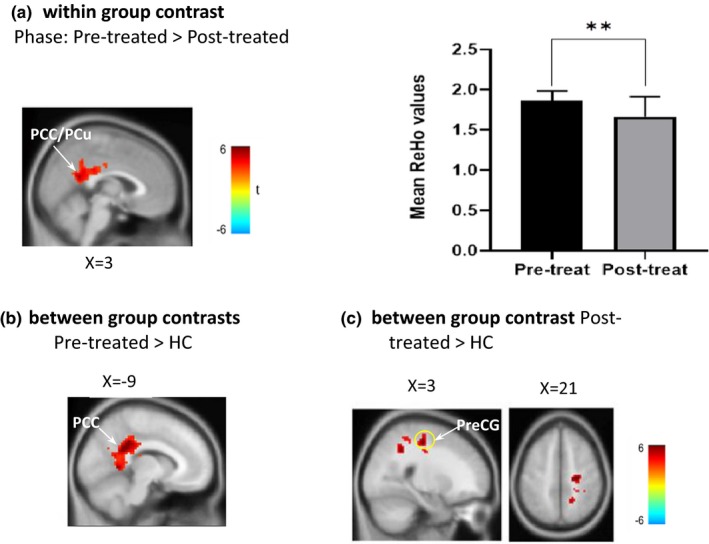
Statistic maps in ReHo analysis of between‐group and between‐phase comparisons. (a) Comparison of patient groups between the pretreated and post‐treated phases. Comparison of the post‐treated sciatica group with the healthy group, showing ReHo in PCC (b) and right precentral gyrus (marked with yellow circle) (c). HC, healthy control; PCC, posterior cingulate cortex; Post‐treated, patients with sciatica after acupuncture treatment; PreCG, precentral gyrus; Pretreated, patients with sciatica before acupuncture treatment; ReHo, regional homogeneity. *Significant differences in between‐phase comparisons, **p < .01

For the major effect of pain for sciatica, between‐group comparison of the pretreated sciatica group and the healthy controls exhibited greater ReHo alternation in the left PCC. For the effect of treatment (within‐group analysis), there was a greater ReHo alteration (Sciatica_pretreatment_ − Sciatica_post‐treatment_) in the coordinate (3, −54, 21; BA 23), which is in the region of the PCC/PCu, according to the MNI template. Compared with healthy controls, patients after acupuncture treatment had increased ReHo alternation in the right precentral gyri (BA 4; Table 2 and Figure 2).

**Table 2:** Comparison of groups: Between‐ and within‐group differences in regional homogeneity

Contrast	Region	BA	Size	*t* score	Coordinate		
					*x*	*y*	*z*
Effect of sciatica (between groups)							
HC > Pretreat	NS						
Pretreat > HC	PCC	23	450	9.94	−9	−42	30
Effect of treatment							
Between groups							
Post‐treat > HC	Precentral gyri	4	193	7.66	24	−21	51
HC > Post‐treat	NS						
Within groups							
Pretreat > Post‐treat	PCC/PCu	23	381	5.21	3	−54	21
Post‐treat > Pretreat	NS						

Note

Peak coordinates refer to Montreal Neurological Institute space. Significance was set at the uncorrected voxel level *p* = .005, followed by the family‐wise error‐corrected cluster level *p* = .05.

Abbreviations: BA, Brodmann area; HC, healthy control group; NS, nonsignificant; PCC, posterior cingulate cortex; Post‐treat, group with sciatica after an acupuncture treatment; Pretreat, group with sciatica before acupuncture treatment.

### 3.4 Interregional FC

**Figure 3:**
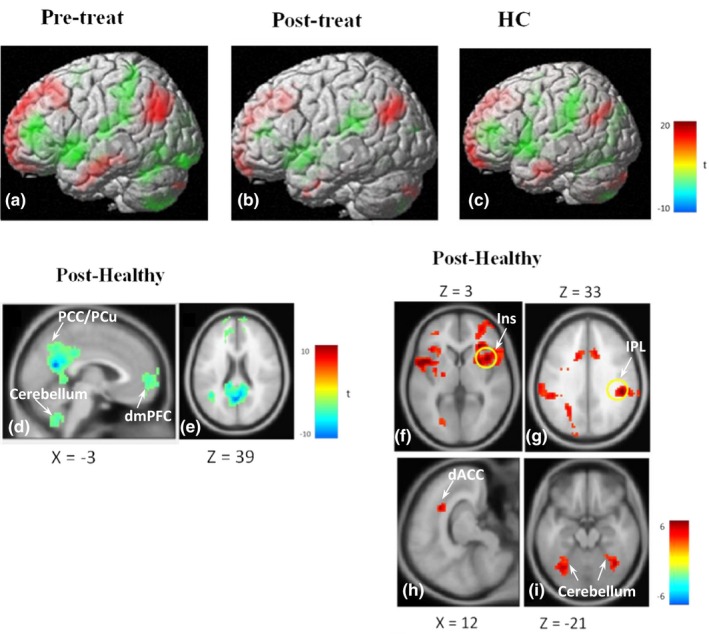
PCC‐seeded FC patterns in the sciatica and healthy groups. DMN of the sciatica group in the pretreated phase (a), post‐treated phase (b) and healthy group (c). Hypoconnectivity between PCC‐seeded FC and left PCu, dmPFC, and cerebellum of the sciatica group after an acupuncture treatment, compared with controls (d, e). Hyperconnectivity with bilateral insula (Ins, marked with a yellow circle in the right), right OFC (f), bilateral IPL (marked with a yellow circle in the right) (g), left dACC (h), and bilateral cerebellum (i). dACC, dorsal anterior cingulate cortex; DMN, default mode network; dmPFC, dorsomedial prefrontal cortex; FC, functional connectivity; Ins, insula; IPL, inferior parietal lobe; OFC, orbitofrontal cortex; PCC, posterior cingulate cortex; PCu, precuneus

We used ReHo results of PCC (*x* = 3, *y* = −54, *z* = 21) as a region of interest (ROI) to examine changes in PCC‐seeded interregional FC. The PCC‐seeded FC exhibited a characteristic DMN pattern of FC in the sciatica and healthy groups (Figure 3a,b). The patterns of DMN and its anticorrelation were visibly different in sciatica patients before and after acupuncture. In addition, the pattern of DMN in the sciatica group with acupuncture treatment was closer to the pattern of the healthy group. When between‐group PCC‐seeded FC maps were compared, there was no significant difference found between the pretreatment sciatica group and the healthy controls. However, after acupuncture treatment, compared to the controls, the sciatica group exhibited hyperconnectivity between PCC‐seeded FC with bilateral insula, bilateral inferior parietal lobules, bilateral cerebellum, right orbitofrontal cortex (OFC, BA 10), and left dorsal anterior cingulate cortex (dACC, BA 32) and exhibited hypoconnectivity between PCC‐seeded FC and left PCu, dmPFC, and cerebellum. In the within‐group analysis, patients in the pre‐acupuncture phase exhibited hyperconnectivity between PCC‐seeded FC and the PCC itself (BA 23; Table 3 and Figure 3).

**Table 3:** Analysis of effects of between‐group and within‐group differences in regional homogeneity (ReHo) seed‐based functional connectivity

Contrast	Region	BA	Size	*t* score	Coordinate		
					*x*	*y*	*z*
Right ReHo seed (3,−54,21)							
Effect of sciatica (between groups)							
HC > AC pre	NS	—	—	—	—	—	—
AC pre > HC	NS						
Effect of acupuncture treatment							
Between groups							
AC post > HC	Insula, right	13	672	7.33	39	12	3
	Insula, left	13	604	6.56	−51	6	3
	OFC, right	10	268	5.27	30	51	6
	IPL, left	40	301	6.34	−54	−42	39
	IPL, right	40	217	6.18	48	−33	33
	dACC	32	318	5.07	12	15	36
	Cerebellum, fusiform, R	—	165	5.83	39	−57	−21
	Cerebellum, fusiform, L	—	486	5.07	−33	−66	−18
AC post < HC	PCu, left	31	1,247	10.56	−3	−60	21
	dmPFC, left‐	9	763	5.00	−15	54	39
	Cerebellum, tonsil	—	222	5.53	6	−54	−48
Within groups							
AC pre > AC post	PCC/PCu, left	23	234	5.69	0	−30	27
AC post > AC pre	NS	—					

Note

Peak coordinates refer to Montreal Neurological Institute space. The significance threshold was set at the uncorrected voxel level *p* = .005, followed by the family‐wise error‐corrected cluster level *p* = .05.

Abbreviations: AC, group of sciatica with acupuncture; dACC, dorsal anterior circulate cortex; dmPFC, dorsomedial prefrontal cortex; HC, group of healthy controls; IPL, inferior parietal lobule; NS, nonsignificant; OFC, orbitofrontal cortex; PCC, posterior cingulate cortex; PCu, precuneus; post, post‐treatment; pre, pretreatment.

### 3.5 Correlation analysis between the FC map and behavior measurements

**Figure 4:**
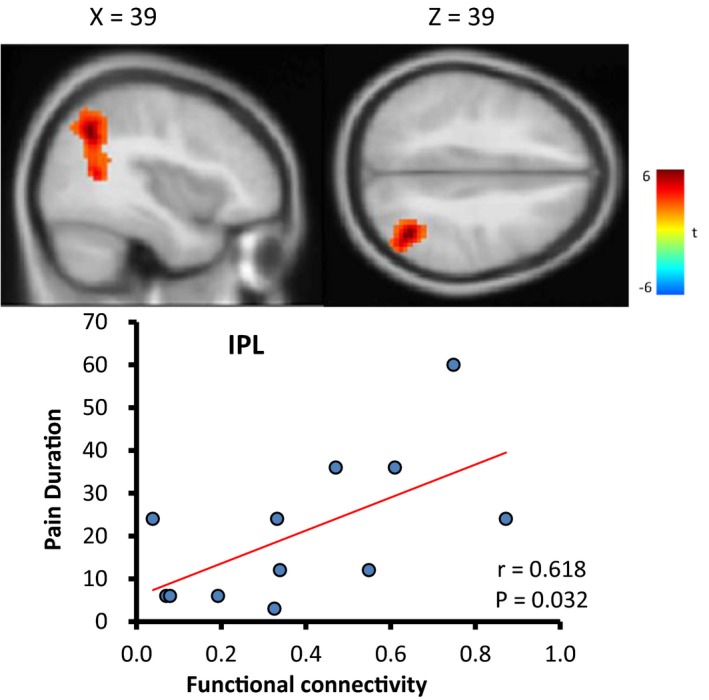
Correlation between the posterior cingulate cortex (PCC)‐seeded functional connectivity (FC) and behaviors in different conditions of sciatica patients

The PCC‐seeded FC was positively correlated with pain duration in the right inferior parietal lobule (IPL) (BA 40; [39, −60, 39]) at baseline (Figure 4). However, PCC‐seeded FC was not significantly correlated with other behavior scores at baseline. After acupuncture treatment, there was no significant correlation between PCC‐FC and any behavior scales.

## 4 DISCUSSION

This preliminary study assessed the clinical effect of acupuncture in treating sciatica and proposed whether acupuncture targeted certain regions of the brain in nonacute/chronic sciatica patients. Through our study, acupuncture may achieve pain relief and resolve disability in patients with sciatica. Seed‐based FC derived from ReHo‐contrast analysis may provide a clue for understanding the central mechanism of acupuncture modulation. We demonstrated that PCC/PCu was a pivotal hub in sciatica treatment with acupuncture. Furthermore, using PCC as an ROI for seed‐based FC whole‐brain analysis, we observed that DMN may play a crucial role in the central analgesic effect of acupuncture. The changes of PCC‐seeded DMN through acupuncture modulation were mainly located in pain‐processing regions such as the bilateral insula, cerebellum, IPL, right mPFC, and dACC. In ReHo, whole‐brain analysis revealed that the precentral gyrus was related to acupuncture modulation. Finally, correlation analysis between PCC‐seeded FC and behavior measurements revealed a positive association with the duration of sciatica in the right IPL prior to acupuncture treatment.

The present findings demonstrate that the nonacute/chronic sciatica group had greater PCC ReHo values than did the healthy control and postacupuncture groups. Higher ReHo values have been reported in several areas of the pain‐related brain network (Shi et al., 2015; Zhao et al., 2013). The PCC in the pain network is altered in chronic pain (Hemington, Wu, Kucyi, Inman, & Davis, 2016; Loggia et al., 2013). The first study of acupuncture on the DMN of healthy individuals indicated that the immediate effect of acupuncture may increase FC of the DMN to pain and affective memory‐related regions of the brain (Dhond et al., 2008). Our findings further addressed that the PCC/PCu was not only an important hub in the DMN and the relevant pain network, but also played a role in the central effect of repeated acupuncture in treating sciatica. In addition, compared with the healthy group, the postacupuncture sciatica group had significantly higher ReHo in the right precentral gyrus. This result is consistent with a previous meta‐analysis study that reported that verum acupuncture stimuli may affect the precentral gyrus relative to resting conditions (Huang et al., 2012). The findings are also consistent with a prior migraine study that reported the precentral gyrus FC of migraine patients was significantly increased after acupuncture treatment (Li et al., 2015). These results suggest that acupuncture may engage in descending pain modulatory systems (DPMS) and the DMN for central analgesia.

Furthermore, we selected PCC based on the contrast of the ReHo map as a ROI to examine the effect of acupuncture analgesia on the DMN. The PCC/PCu plays a crucial role in the DMN (Fransson & Marrelec, 2008). Our results further emphasize that the PCC/PCu is also a key brain region for acupuncture‐treated nonacute/chronic sciatica. The DMN is associated with self‐processing, attention, and memory (Buckner et al., 2008, 2005; Spreng & Grady, 2010) and contributes to acute and chronic pain (Alshelh et al., 2018). However, the exact effects of acupuncture on the DMN for pain relief are unclear. Disruption of the resting state of the DMN in PCC‐FC in the precuneus region has recently been reported in patients with chronic orofacial neuropathic pain (Alshelh et al., 2018). In the present study, there was greater PCC/PCu FC in the sciatica group prior to acupuncture treatment compared to after acupuncture treatment (Figure 3a,b). In addition, compared with the controls, the acupuncture‐treated sciatica group exhibited hyperconnectivity of PCC‐FC in the bilateral insula, bilateral IPL, bilateral cerebellum, right OFC, and left dACC (Figure 3h,i). The changes in resting‐state FC after acupuncture modulation shed light on the central analgesic effect of acupuncture and indicate that these effects may target the DMN and strengthen its connection to pain‐processing areas. An earlier study regarding FC and acupuncture reported that manual acupuncture may reduce activity in pain‐processing regions of the brain in a healthy population during nociceptive stimulation (Hui et al., 2000). A rsfMRI study revealed that the baseline of patients with chronic lower back pain demonstrated greater DMN connectivity to the ACC, left IPL, and right insula, and FC of DMN‐right insula may positively correlate with clinical pain (Loggia et al., 2013). The above regions are consistent with the brain areas affected by acupuncture that were identified in our study. In addition to bilateral insula and bilateral IPL, the patients receiving acupuncture had greater PCC‐seeded FC to the right OFC and dACC, implicating that the analgesic effect of acupuncture may involve emotion, cognition, and reward circuits of the brain (Kringelbach, 2005; Navratilova & Porreca, 2014; Stevens, Hurley, Hurley, & Taber, 2011). Stronger interregional FC between DMN and mPFC was observed in primary dysmenorrhea and migraine patients compared to controls (Coppola et al., 2018; Wu et al., 2016). Similar findings were observed in our study based on visual inspection (Figure 3a). Patients receiving acupuncture treatment had hypoconnectivity of the PCC‐FC to right dmPFC (substrate of ECN network), suggesting that acupuncture could seemingly disintegrate the maladaptive FC between DMN and ECN network for central analgesia through the DPMS (Chen, Spaeth, Spaeth, Retzepi, Ott, & Kong, 2014), alleviating pain rumination (Kucyi et al., 2014; Figure 3b,d,e).

The DMN may be reorganized by chronic pain and exhibits maladaptive connectivity in different types of chronic pain (Baliki et al., 2014). In addition, the cross‐network interaction between the DMN and salience network has been observed in chronic pain. Patients with chronic pain showed less anticorrelated FC between the DMN and salience network compared with that of healthy controls (Hemington et al., 2016). In the present study, the anticorrelation of PCC‐seeded DMN in sciatica patients of pre‐acupuncture was not significantly different than that of healthy controls (Figure 3a,c). However, acupuncture‐treated patients demonstrated global deactivation in the DMN and activation of its anticorrelation (Figure 3b), which is consistent with the results from a task fMRI study demonstrating that acupuncture may mobilize the brain's default mode and its anticorrelated network in healthy controls (Hui et al., 2009). Furthermore, correlation analysis revealed that the PCC‐FC of patients with nonacute sciatica was positively correlated with pain duration in the IPL. This result is similar to previous research on pain scores in chronic pain patients with ankylosing spondylitis which were positively correlated with PCC‐FC with the right temporal junction (Hemington et al., 2016). Collectively, these findings suggest that disrupted DMN–salience network cross‐network connectivity is a major functional aberration in chronic sciatica patients.

There are several limitations to the current study. First, the number of subjects recruited was relatively small, and there was nonsignificant improvement in the quality of life. However, despite the small number of the patients, those treated with acupuncture obtained significantly improved pain relief and decreased disability due to sciatica. Second, we cannot exclude the possibility of a natural course of the disease of the patients. Nevertheless, all twelve patients have received contemporary Western medicine but did not attain a satisfactory therapeutic effect. Furthermore, even sham needles have a placebo effect (Lund, Naslund, Naslund, & Lundeberg, 2009). The therapeutic effects of acupuncture continue over time and cannot be elucidated only in terms of placebo effects (Egorova, Gollub, Gollub, & Kong, 2015; Vickers et al., 2018). We wanted to observe the modulated effect of acupuncture in sciatica treatment; therefore, there was no sham acupuncture group in the present study. Nonetheless, we would like to refine the study design to include a nonacupoint acupuncture group to complete the main trial.

## 5 CONCLUSIONS

In summary, the present study suggests that acupuncture treatment may relieve pain in sciatica patients and normalize its modulation in the relevant brain areas, especially the DMN and DPMS. The endophenotypic changes in brain FC based on acupuncture modulation may provide clues to understand the mechanisms underlying the analgesic effects of acupuncture on chronic pain. Acupuncture may promote robustness in the system (Xu et al., 2018) and normalize the disrupted cross‐network connectivity in chronic pain patients, which could be a useful treatment strategy. More comprehensive, larger‐scale, and randomized controlled studies are necessary to verify these effects.

## CONFLICT OF INTEREST

The authors declare that they have no conflict of interest.

## Data Availability

The data that support the findings of this study are available from the corresponding author upon reasonable request.

## References

[brb31494-bib-0001] Alshelh, Z. , Marciszewski, K. K. , Akhter, R. , Di Pietro, F. , Mills, E. P. , Vickers, E. R. , … Henderson, L. A. (2018). Disruption of default mode network dynamics in acute and chronic pain states. NeuroImage Clinical, 17, 222–231. 10.1016/j.nicl.2017.10.019 29159039 PMC5683191

[brb31494-bib-0002] Andersson, G. B. J. , Pope, M. H. , Frymoyer, J. W. , & Snook, S. (1991) Epidemiology and cost In Pope M. H., Andersson G. B. J., Frymoyer J. W. & Chaffin D. B. (Eds.), Occupational low back pain: Assessment, treatment and retention (pp. 95–113). St Louis, MO: Mosby‐Year Book.

[brb31494-bib-0003] Apkarian, A. V. , Baliki, M. N. , & Geha, P. Y. (2009). Towards a theory of chronic pain. Progress in Neurobiology, 87(2), 81–97. 10.1016/j.pneurobio.2008.09.018 18952143 PMC2650821

[brb31494-bib-0004] Atlas, S. J. , & Nardin, R. A. (2003). Evaluation and treatment of low back pain: An evidence‐based approach to clinical care. Muscle and Nerve, 27(3), 265–284. 10.1002/mus.10311 12635113

[brb31494-bib-0005] Baliki, M. N. , Mansour, A. R. , Baria, A. T. , & Apkarian, A. V. (2014). Functional reorganization of the default mode network across chronic pain conditions. PLoS One, 9(9), e106133 10.1371/journal.pone.0106133 25180885 PMC4152156

[brb31494-bib-0006] Buckner, R. L. , Andrews‐Hanna, J. R. , & Schacter, D. L. (2008). The brain's default network: Anatomy, function, and relevance to disease. Annals of the New York Academy of Sciences, 1124, 1–38. 10.1196/annals.1440.011 18400922

[brb31494-bib-0007] Buckner, R. L. , Snyder, A. Z. , Shannon, B. J. , LaRossa, G. , Sachs, R. , Fotenos, A. F. , … Mintun, M. A. (2005). Molecular, structural, and functional characterization of Alzheimer's disease: Evidence for a relationship between default activity, amyloid, and memory. Journal of Neuroscience, 25(34), 7709–7717. 10.1523/jneurosci.2177-05.2005 16120771 PMC6725245

[brb31494-bib-0008] Cai, R. L. , Shen, G. M. , Wang, H. , & Guan, Y. Y. (2018). Brain functional connectivity network studies of acupuncture: A systematic review on resting‐state fMRI. Journal of Integrative Medicine, 16(1), 26–33. 10.1016/j.joim.2017.12.002 29397089

[brb31494-bib-0009] Cauda, F. , Sacco, K. , Duca, S. , Cocito, D. , D'Agata, F. , Geminiani, G. C. , & Canavero, S. (2009). Altered resting state in diabetic neuropathic pain. PLoS One, 4(2), e4542 10.1371/journal.pone.0004542 19229326 PMC2638013

[brb31494-bib-0010] Chao‐Gan, Y. , & Yu‐Feng, Z. (2010). DPARSF: A MATLAB toolbox for “pipeline” data analysis of resting‐state fMRI. Frontiers in Systems Neuroscience, 4, 13 10.3389/fnsys.2010.00013 20577591 PMC2889691

[brb31494-bib-0011] Chen, X. , Spaeth, R. B. , Freeman, S. G. , Scarborough, D. M. , Hashmi, J. A. , Wey, H.‐Y. , … Kong, J. (2015). The modulation effect of longitudinal acupuncture on resting state functional connectivity in knee osteoarthritis patients. Molecular Pain, 11(1), 67 10.1186/s12990-015-0071-9 26511911 PMC4625557

[brb31494-bib-0012] Chen, X. , Spaeth, R. B. , Retzepi, K. , Ott, D. , & Kong, J. (2014). Acupuncture modulates cortical thickness and functional connectivity in knee osteoarthritis patients. Scientific Reports, 4, 6482 10.1038/srep06482 25258037 PMC4175730

[brb31494-bib-0013] Collins, S. L. , Moore, R. A. , & McQuay, H. J. (1997). The visual analogue pain intensity scale: What is moderate pain in millimetres? Pain, 72(1–2), 95–97. 10.1016/S0304-3959(97)00005-5 9272792

[brb31494-bib-0014] Coppola, G. , Di Renzo, A. , Tinelli, E. , Di Lorenzo, C. , Scapeccia, M. , Parisi, V. , … Pierelli, F. (2018). Resting state connectivity between default mode network and insula encodes acute migraine headache. Cephalalgia, 38(5), 846–854. 10.1177/0333102417715230 28605972

[brb31494-bib-0015] Deyo, R. A. , & Tsui‐Wu, Y. J. (1987). Descriptive epidemiology of low‐back pain and its related medical care in the United States. Spine, 12(3), 264–268. 10.1097/00007632-198704000-00013 2954221

[brb31494-bib-0016] Dhond, R. P. , Yeh, C. , Park, K. , Kettner, N. , & Napadow, V. (2008). Acupuncture modulates resting state connectivity in default and sensorimotor brain networks. Pain, 136(3), 407–418. 10.1016/j.pain.2008.01.011 18337009 PMC2440647

[brb31494-bib-0017] Egorova, N. , Gollub, R. L. , & Kong, J. (2015). Repeated verum but not placebo acupuncture normalizes connectivity in brain regions dysregulated in chronic pain. NeuroImage Clinical, 9, 430–435. 10.1016/j.nicl.2015.09.012 26594625 PMC4596925

[brb31494-bib-0018] Farmer, M. A. , Chanda, M. L. , Parks, E. L. , Baliki, M. N. , Apkarian, A. V. , & Schaeffer, A. J. (2011). Brain functional and anatomical changes in chronic prostatitis/chronic pelvic pain syndrome. Journal of Urology, 186(1), 117–124. 10.1016/j.juro.2011.03.027 21571326 PMC4889821

[brb31494-bib-0019] Fox, M. D. , Zhang, D. , Snyder, A. Z. , & Raichle, M. E. (2009). The global signal and observed anticorrelated resting state brain networks. Journal of Neurophysiology, 101(6), 3270–3283. 10.1152/jn.90777.2008 19339462 PMC2694109

[brb31494-bib-0020] Fransson, P. , & Marrelec, G. (2008). The precuneus/posterior cingulate cortex plays a pivotal role in the default mode network: Evidence from a partial correlation network analysis. NeuroImage, 42(3), 1178–1184. 10.1016/j.neuroimage.2008.05.059 18598773

[brb31494-bib-0021] Grovle, L. , Haugen, A. J. , Keller, A. , Natvig, B. , Brox, J. I. , & Grotle, M. (2010). The bothersomeness of sciatica: Patients' self‐report of paresthesia, weakness and leg pain. European Spine Journal, 19(2), 263–269. 10.1007/s00586-009-1042-5 19488793 PMC2899809

[brb31494-bib-0022] Grovle, L. , Haugen, A. J. , Natvig, B. , Brox, J. I. , & Grotle, M. (2013). The prognosis of self‐reported paresthesia and weakness in disc‐related sciatica. European Spine Journal, 22(11), 2488–2495. 10.1007/s00586-013-2871-9 23771579 PMC3886504

[brb31494-bib-0023] Hemington, K. S. , Wu, Q. , Kucyi, A. , Inman, R. D. , & Davis, K. D. (2016). Abnormal cross‐network functional connectivity in chronic pain and its association with clinical symptoms. Brain Structure and Function, 221(8), 4203–4219. 10.1007/s00429-015-1161-1 26669874

[brb31494-bib-0024] Huang, W. , Pach, D. , Napadow, V. , Park, K. , Long, X. , Neumann, J. , … Witt, C. M. (2012). Characterizing acupuncture stimuli using brain imaging with fMRI ‐ A systematic review and meta‐analysis of the literature. PLoS One, 7(4), e32960 10.1371/journal.pone.0032960 22496739 PMC3322129

[brb31494-bib-0025] Hui, K. K. S. , Liu, J. , Makris, N. , Gollub, R. L. , Chen, A. J. W. , I. Moore, C. , … Kwong, K. K. (2000). Acupuncture modulates the limbic system and subcortical gray structures of the human brain: Evidence from fMRI studies in normal subjects. Human Brain Mapping, 9(1), 13–25. 10.1002/(SICI)1097-0193(2000)9:1<13:AID-HBM2>3.0.CO;2-F 10643726 PMC6871878

[brb31494-bib-0026] Hui, K. K. S. , Marina, O. , Claunch, J. D. , Nixon, E. E. , Fang, J. , Liu, J. , … Rosen, B. R. (2009). Acupuncture mobilizes the brain's default mode and its anti‐correlated network in healthy subjects. Brain Research, 1287, 84–103. 10.1016/j.brainres.2009.06.061 19559684 PMC3742122

[brb31494-bib-0027] Huskisson, E. C. (1974). Measurement of pain. Lancet, 2(7889), 1127–1131. 4139420 10.1016/s0140-6736(74)90884-8

[brb31494-bib-0028] Jia, B. , Liu, Z. , Min, B. , Wang, Z. , Zhou, A. , Li, Y. , … Jia, J. (2015). The effects of acupuncture at real or sham acupoints on the intrinsic brain activity in mild cognitive impairment patients. Evidence‐Based Complementary and Alternative Medicine, 2015, 529675 10.1155/2015/529675 26064166 PMC4433670

[brb31494-bib-0029] Jiang, L. , & Zuo, X. N. (2016). Regional homogeneity: A multimodal, multiscale neuroimaging marker of the human connectome. Neuroscientist, 22(5), 486–505. 10.1177/1073858415595004 26170004 PMC5021216

[brb31494-bib-0030] Kim, M. , Guilfoyle, M. R. , Seeley, H. M. , & Laing, R. J. (2010). A modified Roland–Morris disability scale for the assessment of sciatica. Acta Neurochirurgica, 152(9), 1549–1553. 10.1007/s00701-010-0679-5 20467761

[brb31494-bib-0031] Kringelbach, M. L. (2005). The human orbitofrontal cortex: Linking reward to hedonic experience. Nature Reviews Neuroscience, 6(9), 691–702. 10.1038/nrn1747 16136173

[brb31494-bib-0032] Kucyi, A. , & Davis, K. D. (2015). The dynamic pain connectome. Trends in Neurosciences, 38(2), 86–95. 10.1016/j.tins.2014.11.006 25541287

[brb31494-bib-0033] Kucyi, A. , Moayedi, M. , Weissman‐Fogel, I. , Goldberg, M. B. , Freeman, B. V. , Tenenbaum, H. C. , & Davis, K. D. (2014). Enhanced medial prefrontal‐default mode network functional connectivity in chronic pain and its association with pain rumination. Journal of Neuroscience, 34(11), 3969–3975. 10.1523/jneurosci.5055-13.2014 24623774 PMC6705280

[brb31494-bib-0034] Lewis, R. A. , Williams, N. H. , Sutton, A. J. , Burton, K. , Din, N. U. , Matar, H. E. , … Wilkinson, C. (2015). Comparative clinical effectiveness of management strategies for sciatica: Systematic review and network meta‐analyses. Spine Journal, 15(6), 1461–1477. 10.1016/j.spinee.2013.08.049 24412033

[brb31494-bib-0035] Li, K. , Zhang, Y. , Ning, Y. , Zhang, H. , Liu, H. , Fu, C. , … Zou, Y. (2015). The effects of acupuncture treatment on the right frontoparietal network in migraine without aura patients. The Journal of Headache and Pain, 16, 518 10.1186/s10194-015-0518-4 25916336 PMC4411327

[brb31494-bib-0036] Liu, C.‐H. , & Chen, F.‐P. (2017). Therapeutic approach of acupuncture for sciatica: A brief review. Neuropsychiatry, 7(2), 149–155.

[brb31494-bib-0037] Loggia, M. L. , Kim, J. , Gollub, R. L. , Vangel, M. G. , Kirsch, I. , Kong, J. , … Napadow, V. (2013). Default mode network connectivity encodes clinical pain: An arterial spin labeling study. Pain, 154(1), 24–33. 10.1016/j.pain.2012.07.029 23111164 PMC3534957

[brb31494-bib-0038] Luijsterburg, P. A. , Verhagen, A. P. , Ostelo, R. W. , van Os, T. A. , Peul, W. C. , & Koes, B. W. (2007). Effectiveness of conservative treatments for the lumbosacral radicular syndrome: A systematic review. European Spine Journal, 16(7), 881–899. 10.1007/s00586-007-0367-1 17415595 PMC2219647

[brb31494-bib-0039] Lund, I. , Naslund, J. , & Lundeberg, T. (2009). Minimal acupuncture is not a valid placebo control in randomised controlled trials of acupuncture: A physiologist's perspective. Chinese Medicine, 4, 1 10.1186/1749-8546-4-1 19183454 PMC2644695

[brb31494-bib-0040] May, A. (2008). Chronic pain may change the structure of the brain. Pain, 137(1), 7–15. 10.1016/j.pain.2008.02.034 18410991

[brb31494-bib-0041] Murphy, K. , Birn, R. M. , Handwerker, D. A. , Jones, T. B. , & Bandettini, P. A. (2009). The impact of global signal regression on resting state correlations: Are anti‐correlated networks introduced? NeuroImage, 44(3), 893–905. 10.1016/j.neuroimage.2008.09.036 18976716 PMC2750906

[brb31494-bib-0042] Murray, C. J. , & Lopez, A. D. (2013). Measuring the global burden of disease. New England Journal of Medicine, 369(5), 448–457. 10.1056/NEJMra1201534 23902484

[brb31494-bib-0043] Navratilova, E. , & Porreca, F. (2014). Reward and motivation in pain and pain relief. Nature Neuroscience, 17(10), 1304–1312. 10.1038/nn.3811 25254980 PMC4301417

[brb31494-bib-0044] Peul, W. C. , van Houwelingen, H. C. , van den Hout, W. B. , Brand, R. , Eekhof, J. A. H. , Tans, J. T. J. , … Koes, B. W. (2007). Surgery versus prolonged conservative treatment for sciatica. New England Journal of Medicine, 356(22), 2245–2256. 10.1056/NEJMoa064039 17538084

[brb31494-bib-0045] Pinto, R. Z. , Maher, C. G. , Ferreira, M. L. , Ferreira, P. H. , Hancock, M. , Oliveira, V. C. , … Koes, B. (2012). Drugs for relief of pain in patients with sciatica: Systematic review and meta‐analysis. BMJ, 344, e497 10.1136/bmj.e497 22331277 PMC3278391

[brb31494-bib-0046] Qin, Z. , Liu, X. , Wu, J. , Zhai, Y. , & Liu, Z. (2015). Effectiveness of acupuncture for treating sciatica: A systematic review and meta‐analysis. Evidence‐Based Complementary and Alternative Medicine, 2015, 425108 10.1155/2015/425108 26576192 PMC4631886

[brb31494-bib-0047] Raichle, M. E. (2015). The brain's default mode network. Annual Review of Neuroscience, 38, 433–447. 10.1146/annurev-neuro-071013-014030 25938726

[brb31494-bib-0048] Roland, M. , & Morris, R. (1983). A study of the natural history of back pain. Part I: Development of a reliable and sensitive measure of disability in low‐back pain. Spine, 8(2), 141–144. 6222486 10.1097/00007632-198303000-00004

[brb31494-bib-0049] Shi, Y. , Liu, Z. , Zhang, S. , Li, Q. , Guo, S. , Yang, J. , & Wu, W. (2015). Brain network response to acupuncture stimuli in experimental acute low back pain: An fMRI study. Evidence‐Based Complementary and Alternative Medicine, 2015, 210120 10.1155/2015/210120 26161117 PMC4487721

[brb31494-bib-0050] Spreng, R. N. , & Grady, C. L. (2010). Patterns of brain activity supporting autobiographical memory, prospection, and theory of mind, and their relationship to the default mode network. Journal of Cognitive Neuroscience, 22(6), 1112–1123. 10.1162/jocn.2009.21282 19580387

[brb31494-bib-0051] Stevens, F. L. , Hurley, R. A. , & Taber, K. H. (2011). Anterior cingulate cortex: Unique role in cognition and emotion. Journal of Neuropsychiatry and Clinical Neurosciences, 23(2), 121–125. 10.1176/appi.neuropsych.23.2.12110.1176/jnp.23.2.jnp121 21677237

[brb31494-bib-0052] Valat, J. P. , Genevay, S. , Marty, M. , Rozenberg, S. , & Koes, B. (2010). Sciatica. Best Practice & Research Clinical Rheumatology, 24(2), 241–252. 10.1016/j.berh.2009.11.005 20227645

[brb31494-bib-0053] Vickers, A. J. , Vertosick, E. A. , Lewith, G. , MacPherson, H. , Foster, N. E. , Sherman, K. J. , … Linde, K. (2018). Acupuncture for chronic pain: Update of an individual patient data meta‐analysis. The Journal of Pain, 19(5), 455–474. 10.1016/j.jpain.2017.11.005 29198932 PMC5927830

[brb31494-bib-0054] Videman, T. , Nurminen, T. , Tola, S. , Kuorinka, I. , Vanharanta, H. , & Troup, J. D. G. (1984). Low‐back pain in nurses and some loading factors of work. Spine, 9(4), 400–404. 10.1097/00007632-198405000-00013 6236565

[brb31494-bib-0055] Wu, T.‐H. , Tu, C.‐H. , Chao, H.‐T. , Li, W.‐C. , Low, I. , Chuang, C.‐Y. , … Hsieh, J.‐C. (2016). Dynamic changes of functional pain connectome in women with primary dysmenorrhea. Scientific Reports, 6, 24543 10.1038/srep24543 27089970 PMC4835697

[brb31494-bib-0056] Xu, Y. , Guo, Y. , Song, Y. , Zhang, K. , Zhang, Y. , Li, Q. , … Guo, Y. I. (2018). A new theory for acupuncture: Promoting robust regulation. Journal of Acupuncture and Meridian Studies, 11(1), 39–43. 10.1016/j.jams.2017.11.004 29482800

[brb31494-bib-0057] Yan, F. X. , Wu, C. W. , Cheng, S. Y. , Lim, K. E. , Hsu, Y. Y. , & Liu, H. L. (2013). Resting‐state functional magnetic resonance imaging analysis with seed definition constrained by regional homogeneity. Brain Connectivity, 3(4), 438–449. 10.1089/brain.2013.0164 23802999

[brb31494-bib-0058] Yao, G. , & Wu, C. H. (2005). Factorial invariance of the WHOQOL‐BREF among disease groups. Quality of Life Research, 14(8), 1881–1888. 10.1007/s11136-005-3867-7 16155775

[brb31494-bib-0059] Zang, Y. , Jiang, T. , Lu, Y. , He, Y. , & Tian, L. (2004). Regional homogeneity approach to fMRI data analysis. NeuroImage, 22(1), 394–400. 10.1016/j.neuroimage.2003.12.030 15110032

[brb31494-bib-0060] Zhang, J. , Cai, X. , Wang, Y. , Zheng, Y. U. , Qu, S. , Zhang, Z. , … Huang, Y. (2019). Different brain activation after acupuncture at combined acupoints and single acupoint in hypertension patients: An Rs‐fMRI study based on ReHo analysis. Evidence‐Based Complementary and Alternative Medicine, 2019, 5262896 10.1155/2019/5262896 30719061 PMC6335668

[brb31494-bib-0061] Zhang, J. , Zheng, Y. U. , Wang, Y. , Qu, S. , Zhang, S. , Wu, C. , … Huang, Y. (2016). Evidence of a synergistic effect of acupoint combination: A resting‐state functional magnetic resonance imaging study. Journal of Alternative and Complementary Medicine, 22(10), 800–809. 10.1089/acm.2016.0016 27548054 PMC5067799

[brb31494-bib-0062] Zhao, L. , Liu, J. , Dong, X. , Peng, Y. , Yuan, K. , Wu, F. , … Liang, F. (2013). Alterations in regional homogeneity assessed by fMRI in patients with migraine without aura stratified by disease duration. The Journal of Headache and Pain, 14, 85 10.1186/1129-2377-14-85 24134520 PMC3853130

